# Golden Hours in Disaster Response and Early Clinical Outcomes: An Analysis of the First 24 Hours of the Kahramanmaraş Earthquake

**DOI:** 10.3390/jcm15218366

**Published:** 2026-10-27

**Authors:** Ahmet Yeşil, Mahmut Yaman

**Affiliations:** Emergency Medicine Department, Faculty of Medicine, Dicle University, Diyarbakır 21280, Türkiye; drmahmutyaman@hotmail.com

**Keywords:** earthquake, crush syndrome, rhabdomyolysis, golden hour, trauma severity

## Abstract

**Background**: This study examined the association between admission time within 24 h of the Kahramanmaraş earthquakes and trauma severity, rhabdomyolysis, and in-hospital mortality, and its potential as a temporal reference for field triage in mass-casualty incidents. **Methods**: This retrospective study included earthquake-related injury patients who presented to our tertiary emergency department within 24 h of the earthquake onset (04:17, 6 February 2023). Non-survivors who died before diagnostic work-up, and therefore had no laboratory results or trauma scores, were excluded from the analysis. Patients were compared by admission within the golden hour (≤6 h) or later (>6 h); laboratory samples were obtained within the first hour of presentation. Mortality predictors were examined using univariate and multivariable logistic regression, and receiver operating characteristic (ROC) analysis evaluated admission time for predicting rhabdomyolysis. **Results**: Three of 63 patients (4.8%) died; lower Glasgow Coma Scale scores and higher trauma severity indices were associated with mortality, whereas admission time and entrapment were not (very few deaths). Patients admitted after 6 h had higher Abbreviated Injury Scale (AIS) grades and significantly higher creatine kinase (CK), creatine kinase-MB isoenzyme (CK-MB), lactate dehydrogenase (LDH), C-reactive protein (CRP), and international normalized ratio (INR) values and longer hospitalizations. Admission time predicted rhabdomyolysis well (area under the curve [AUC] = 0.840; cutoff: 9 h) and correlated positively with log-transformed CK (r = 0.733). **Conclusions**: The initial temporal window may be associated with muscle and organ injury severity among patients reaching hospital, but does not support redefining the golden hour as a survival determinant across the care continuum. The time threshold is hypothesis-generating and requires external validation before field triage use.

## 1. Introduction

Earthquakes are natural disasters that place severe strain on health systems, as large numbers of patients require emergency medical care within a short period of time. When individuals are compressed beneath collapsed structures for extended periods, crush syndrome—a form of traumatic rhabdomyolysis—emerges as a significant cause of mortality in earthquake situations [1]. The pathophysiology involves prolonged compression of skeletal muscle, leading to severe systemic complications including cardiovascular instability, electrolyte imbalances, metabolic derangements, and renal dysfunction [2]. As a result, crush syndrome ranks among the leading causes of earthquake-related death, trailing only direct trauma itself [3].

Southeastern Türkiye was struck on 6 February 2023 by a pair of major earthquakes—magnitude 7.6 and 7.7—centered near Kahramanmaraş, leaving 11 provinces in ruin. Tens of thousands of casualties overwhelmed the region’s emergency medical capacity [3]. Among survivors, orthopedic injuries dominated the clinical picture, with crush trauma and compartment syndrome standing out as the most commonly managed surgical emergencies [3,4].

The phrase “golden hour” has entered the trauma literature to capture how decisively early post-injury intervention shapes patient survival. The underlying premise holds that definitive resuscitative care delivered within an injury’s first 60 min can substantially cut mortality risk. This is borne out in studies of non-compressible torso hemorrhage, where longer prehospital intervals—particularly beyond the first 30 min—correspond to sharply elevated mortality [5]. Taken together, this evidence explains the premium placed on swift transport and immediate intervention when managing patients with severe trauma.

The pathological cascade in crush syndrome involves prolonged ischemia of compressed muscle tissue. Once the compressive force is relieved and blood flow resumes, affected muscle cells spill their intracellular contents—myoglobin, creatine kinase (CK), and various electrolytes—into the bloodstream [2]. Among these markers, serum CK elevation serves as a primary indicator of myofibrillar damage and its magnitude correlates with the risk of acute kidney injury development [6]. The concurrent electrolyte derangements, particularly hyperkalemia and hypocalcemia, create additional hazards through cardiac dysrhythmia risk, substantially increasing morbidity in the acute phase [2].

Among crush injury patients evaluated after the Kahramanmaraş earthquake, elevated CK and base deficit values correlated significantly with extremity injury severity, dialysis requirement, and mortality risk [6].

A multicenter retrospective analysis stratifying patients by crush syndrome severity found markedly higher complication rates among severe/critical cases—spanning arrhythmia, disseminated intravascular coagulation, sepsis, and acute respiratory distress—together with greater intensive care requirements and poorer survival [4]. The same study tied longer periods of rubble entrapment to more severe crush syndrome presentations.

Comparing crush syndrome patients who did versus did not develop acute kidney injury after the same earthquake, one investigation found that those who developed kidney injury had experienced longer entrapment periods and higher CK and myoglobin concentrations, together with greater hemodialysis needs and prolonged hospitalization [7].

Analysis of this same patient population highlighted early diagnosis, aggressive fluid resuscitation, and coordinated multidisciplinary care as pivotal determinants of improved outcomes among patients presenting with combined crush syndrome and renal involvement [8].

While previous reports have extensively characterized laboratory–outcome relationships in patients with crush syndrome following the Kahramanmaraş earthquakes, few studies have simultaneously examined how hospital admission time, viewed through the golden hour concept, relates to trauma severity, the onset of rhabdomyolysis, and early mortality. Therefore, the aim of this study was to characterize the clinical and laboratory profiles of patients admitted to our tertiary emergency department within the first 24 h after the earthquake and to evaluate the association of admission timing with trauma severity, rhabdomyolysis development, and survival. As a secondary, exploratory objective, we investigated whether admission time could serve as a quantitative, threshold-based predictor of rhabdomyolysis, with the intent of generating hypotheses for future field triage protocols in mass-casualty earthquake responses.

## 2. Materials and Methods

### 2.1. Study Design and Setting

This retrospective, observational investigation drew on clinical records gathered from an earthquake-affected region. Our institutional base was Dicle University Faculty of Medicine Hospital in Diyarbakır, a tertiary referral center strategically positioned in Southeastern Anatolia roughly 150 km north of Kahramanmaraş. Given this proximity and the region’s healthcare infrastructure, our facility absorbed mass casualty referrals from several neighboring provinces throughout the first 24 h after the 6 February 2023 earthquakes. The first earthquake struck at 04:17 on 6 February 2023, and this moment was defined as time zero of the study. We retrospectively reviewed the electronic hospital and emergency department records spanning the 24-h interval from earthquake onset (04:17 on 6 February) through to 04:17 on 7 February 2023. Admission time was defined as the interval, in hours, between earthquake onset and the patient’s arrival at our emergency department.

### 2.2. Study Population

**Figure 1:**
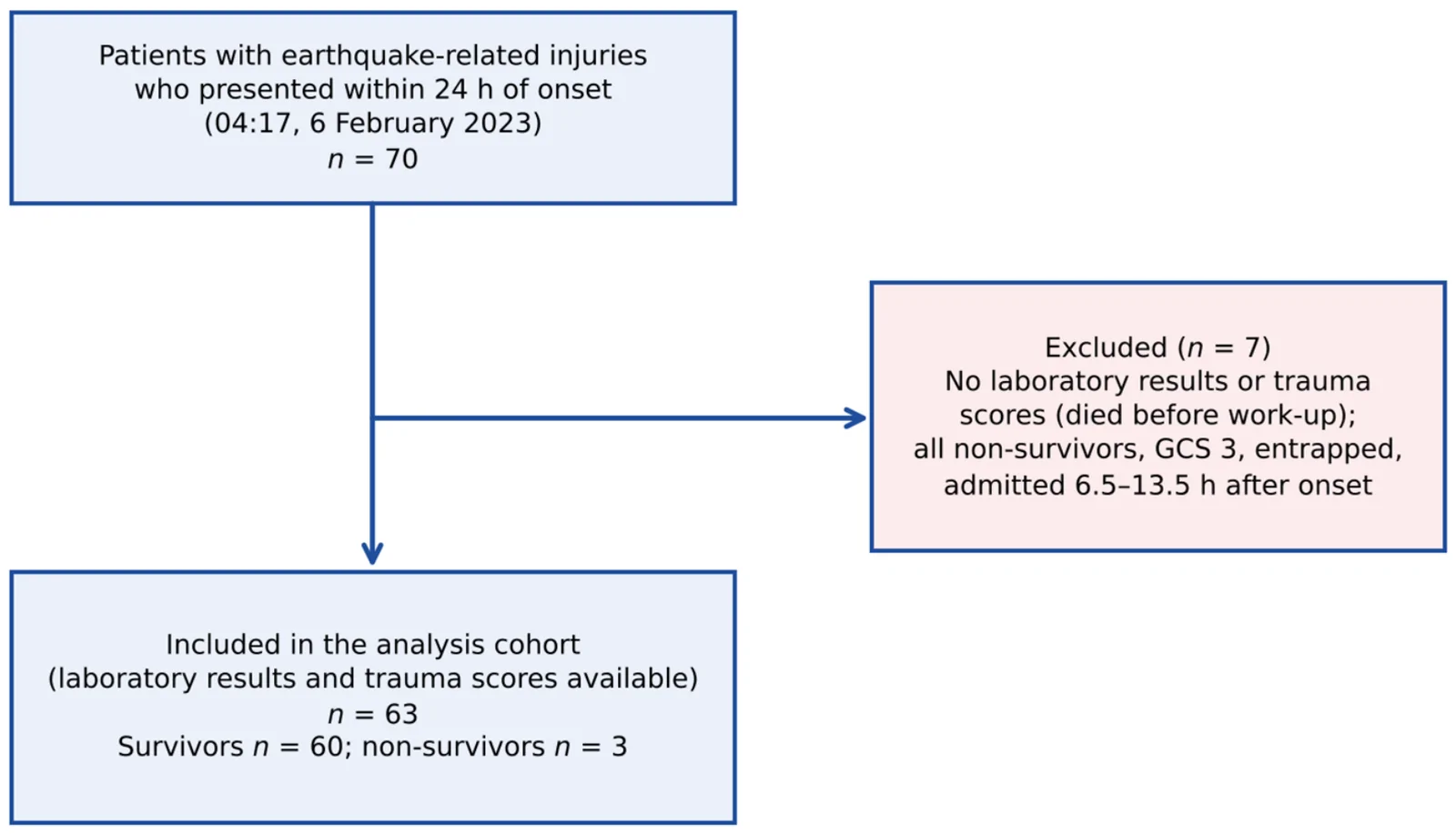
Flow diagram of patient selection. Of the 70 patients with earthquake-related injuries who presented within 24 h of onset (04:17, 6 February 2023), 7 non-survivors without laboratory results or trauma scores were excluded, leaving 63 patients (60 survivors and 3 non-survivors) in the analysis cohort.

All patients who presented to our emergency department within the designated 24-h post-earthquake window (04:17 on 6 February to 04:17 on 7 February 2023) with a physician-confirmed earthquake-related injury or an associated clinical syndrome (e.g., crush injury, respiratory distress, hypothermia, hypovolemic shock, or limb ischemia) were included, with no restriction on age. Presentations unrelated to the earthquake itself (such as cardiac events or diabetic ketoacidosis) were not eligible. Seventy eligible patients were identified. Seven patients who died before the diagnostic work-up could be completed, and for whom no laboratory results or trauma scores (Injury Severity Score [ISS], New Injury Severity Score [NISS], and Revised Trauma Score [RTS]) were available, were excluded; the remaining 63 patients (60 survivors and 3 non-survivors) formed the analysis cohort (Figure 1). All seven excluded patients were non-survivors with a Glasgow Coma Scale (GCS) score of 3 who had been trapped under rubble and had been admitted between 6.5 and 13.5 h after the earthquake onset.

### 2.3. Data Collection

Data extraction followed a standardized collection instrument populated through review of the hospital electronic medical record system alongside emergency department admission logs. Recorded variables spanned demographic details (age, sex, admission time), trauma mechanism (rubble entrapment, escape-related injury, or non-traumatic cause), vital signs at presentation, GCS scores, trauma type (blunt versus penetrating), the Abbreviated Injury Scale (AIS) grade recorded for each patient, rhabdomyolysis-related laboratory findings, and in-hospital outcome. Patients reached the hospital either by 112 emergency ambulance or by their own means; the mode of transport was not systematically documented in the records and could not be analyzed. Two investigators cross-checked all entries independently, resolving discrepancies by joint consensus review.

### 2.4. Variables and Definitions

Vital signs at presentation—arterial pressure, pulse, respiratory rate, oxygen saturation, and core temperature—were systematically documented. To quantify trauma severity objectively, we calculated the ISS, NISS, and RTS; ISS and NISS are derived from AIS severity grades. In addition, the patient-level AIS grade (0 = no injury; 1 = minor to 6 = maximal) was analyzed as an ordinal measure of anatomic injury severity. Blood samples were obtained within the first hour after presentation to the emergency department. The laboratory workup covered blood gas parameters (pH, lactate, base excess), renal indices (creatinine and urea), muscle injury markers (CK, creatine kinase-MB isoenzyme [CK-MB], lactate dehydrogenase [LDH]), inflammatory markers (C-reactive protein [CRP] and white cell count), and coagulation studies (international normalized ratio [INR] and D-dimer). A diagnosis of rhabdomyolysis required both CK elevation and a compatible clinical presentation. Patients were categorized as admitted within the golden hour (≤6 h) or later (>6 h). The outcome of interest was in-hospital mortality.

### 2.5. Ethical Considerations

This work was carried out in keeping with the ethical principles of the Declaration of Helsinki, having received approval from the relevant institutional ethics board (approval date: 19 February 2025; approval number: 105). Patient records were fully anonymized before analysis to preserve confidentiality, and all data were used solely for research purposes.

Because of its retrospective design, this study was granted a waiver of individual informed consent by the ethics committee.

### 2.6. Statistical Analysis

Data analysis was performed using IBM SPSS Statistics, version 27.0 (IBM Corp., Armonk, NY, USA); Firth penalized-likelihood logistic regression was performed in Python (version 3.12). The Shapiro–Wilk test was used to assess the normality of continuous variables. Continuous variables are reported as medians with interquartile ranges (IQRs; 25th–75th percentiles), and categorical variables as counts and percentages. Missing data were handled by available-case analysis, and no imputation was performed. Because excluding non-survivors without laboratory data may bias mortality estimates, the characteristics of the excluded patients are reported descriptively.

Between-group comparisons of continuous variables were performed using the Mann–Whitney U test, and categorical variables were compared using Fisher’s exact test. Patients were compared according to golden-hour admission status. Factors associated with in-hospital mortality were evaluated by univariate logistic regression, with odds ratios (ORs) and 95% confidence intervals (CIs). Multivariable logistic regression models were fitted using Firth’s penalized likelihood method, with 95% confidence intervals and *p* values based on the profile penalized likelihood. Because only three deaths occurred in the analysis cohort, each model was limited to two covariates and all multivariable estimates are considered exploratory.

The discriminative ability of admission time for rhabdomyolysis was assessed by receiver operating characteristic (ROC) curve analysis; the area under the curve (AUC) and its 95% CI were estimated through 3000 bootstrap iterations, and the optimal cutoff was determined using the Youden index. The relationship between CK and admission time was assessed using the Pearson correlation coefficient and simple linear regression on log-transformed CK values, with the coefficient of determination (R^2^) used to gauge model fit.

All statistical tests were two-tailed, and a *p*-value below 0.05 was considered statistically significant.

## 3. Results

### 3.1. Patient Characteristics and Mortality

Of the 70 eligible patients, 7 non-survivors without laboratory results or trauma scores were excluded (Figure 1), leaving 63 patients for analysis; 3 of these (4.8%) died during hospitalization. Non-survivors showed markedly worse consciousness (GCS; *p* < 0.001), higher AIS grade (median 4.0 [IQR 3.0–4.0] vs. 2.0 [1.0–3.0]; *p* = 0.049), higher ISS (*p* = 0.006), higher NISS (*p* = 0.008), and lower RTS (*p* = 0.002) scores, and more frequently required dialysis (2/3 vs. 2/60; *p* = 0.009) and intensive care (3/3 vs. 17/60; *p* = 0.029). No meaningful group differences emerged for age, sex, admission time, rubble entrapment (66.7% vs. 50.0%; *p* = 1.000), or the laboratory markers assessed (lactate, CK, creatinine, CRP; all *p* > 0.05) (Table 1); these comparisons are limited by the very small number of deaths. The 7 excluded patients were all non-survivors with a GCS of 3 who had been trapped under rubble; their median admission time was 13.5 h (range 6.5–13.5), their ages ranged from 10 to 45 years (median 13), and their length of stay was 0 days. Laboratory results and trauma scores were available for all 63 analyzed patients (60 survivors and 3 non-survivors); CK-MB was available for 53 patients and rhabdomyolysis status for 62, and comparisons were based on the available data. Injury severity by AIS grade in the analysis cohort was 0 in 2 patients, 1 in 21, 2 in 19, 3 in 16, and 4 in 5 (median 2.0 [IQR 1.0–3.0]); 21 patients (33.3%) had an AIS grade of ≥3 (2/3 non-survivors vs. 19/60 survivors).

### 3.2. Admission Time, Trauma Severity, and Laboratory Findings

Patients who arrived more than 6 h post-earthquake had steeper trauma severity indices than earlier arrivals—AIS grade rose from 1.0 to 2.0 (*p* = 0.004), ISS climbed from 4.0 to 9.0 (*p* = 0.017) and NISS from 4.0 to 10.0 (*p* = 0.004), whereas GCS did not differ between the groups (*p* = 0.442; median 15.0 in both groups). This delayed group also showed substantially higher CK (250.1 to 6219.4 U/L; *p* < 0.001), along with elevated CK-MB (*p* = 0.009), LDH (*p* = 0.003), CRP (*p* < 0.001), and INR (*p* = 0.007), plus a longer hospital stay (0.0 to 8.7 days; *p* < 0.001) (Table 2).

### 3.3. Admission Time as a Predictor of Rhabdomyolysis

**Figure 2:**
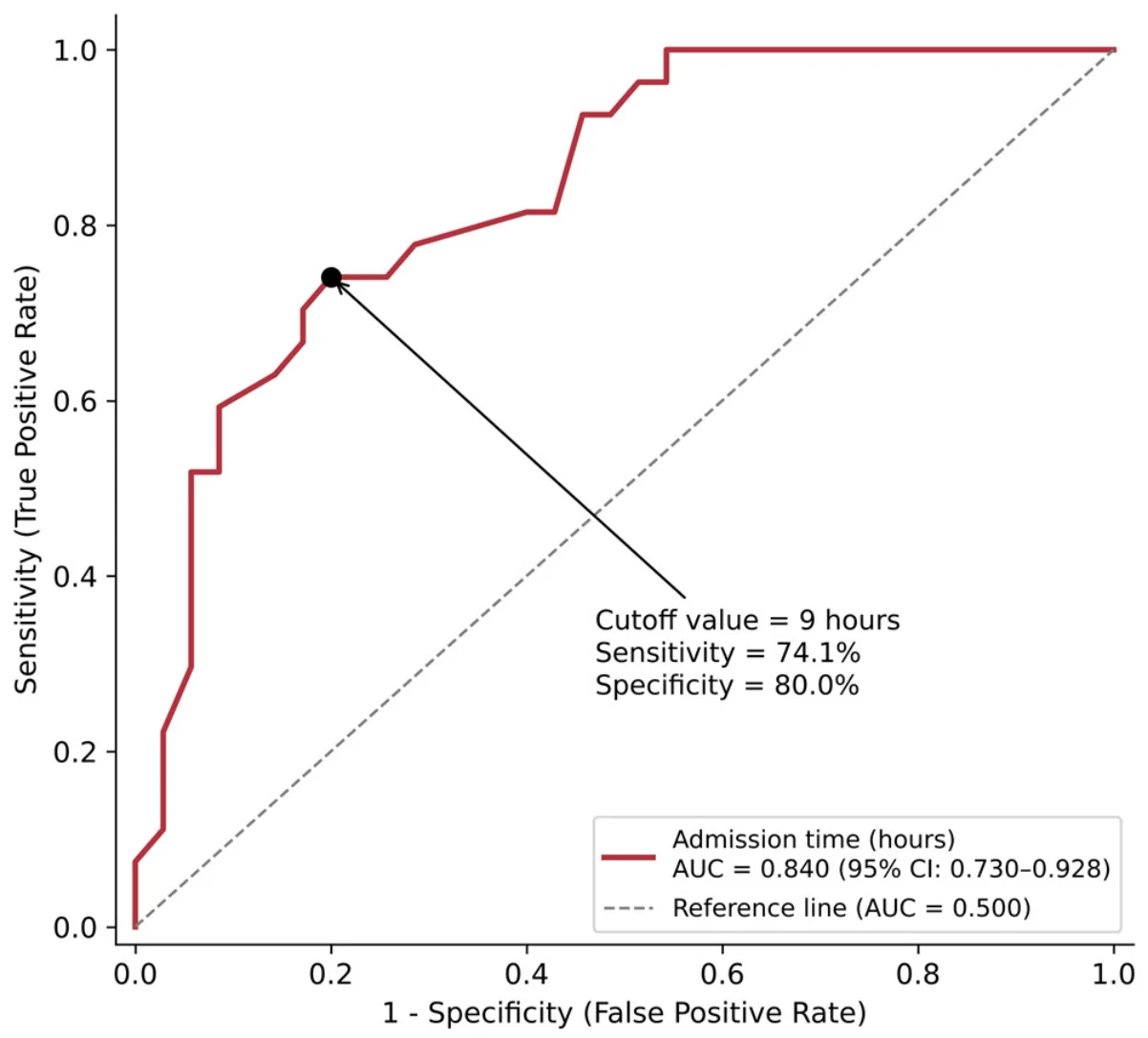
Predictive Accuracy of Admission Time for Rhabdomyolysis (ROC Analysis): ROC—Receiver Operating Characteristic Curve; AUC—Area Under the Curve; CI—Confidence Interval.

Assessing admission time as a predictor of rhabdomyolysis produced an AUC of 0.840 (95% CI 0.730–0.928; *p* < 0.001), reflecting good discriminative ability. The Youden index pointed to 9 h as the optimal cutoff, corresponding to 74.1% sensitivity and 80.0% specificity (Figure 2).

### 3.4. Factors Associated with In-Hospital Mortality

In univariate logistic regression, each GCS point gained lowered mortality odds (0.61; 95% CI 0.44–0.84; *p* = 0.003), while each ISS point raised them (1.17; 95% CI 1.02–1.35; *p* = 0.022). Rubble entrapment was not significantly associated with mortality (OR 2.00; 95% CI 0.17–23.25; *p* = 0.580). Admission time was not associated with mortality when analyzed as a continuous variable (*p* = 0.332). However, admission time was closely related to entrapment (median 17.3 [IQR 6.4–20.0] hours in trapped vs. 4.0 [1.2–6.0] hours in non-trapped patients; *p* < 0.001), and crude in-hospital mortality did not differ significantly between patients admitted beyond 6 h and earlier arrivals (2/31, 6.5% vs. 1/32, 3.1%; *p* = 0.613). To account for this interdependence, multivariable models were fitted, each limited to two covariates because only three deaths occurred. After adjustment for entrapment (Model 1), admission time was not independently associated with mortality (adjusted OR 1.06 per hour; 95% CI 0.89–1.34; *p* = 0.528), and entrapment was likewise not independently associated with mortality (adjusted OR 1.02; 95% CI 0.02–18.70; *p* = 0.988). When admission time was dichotomized at 6 h and entered with entrapment, arrival beyond 6 h was likewise not independently associated with mortality (adjusted OR 1.48; 95% CI 0.13–24.23; *p* = 0.763). In Model 2, adjusted for GCS, admission time was not significantly associated with mortality (adjusted OR 1.22 per hour; 95% CI 0.99–103.26; *p* = 0.068), whereas lower GCS remained strongly associated with mortality (adjusted OR 0.55 per point; 95% CI <0.01–0.78; *p* < 0.001). In Model 3, adjusted for ISS, which was available for all analyzed patients, admission time was not associated with mortality (adjusted OR 1.02 per hour; 95% CI 0.85–1.22; *p* = 0.850), whereas ISS remained associated with mortality (adjusted OR 1.12 per point; 95% CI 1.04–1.29; *p* = 0.001) (Table 3).

### 3.5. Relationship Between Admission Time and Serum CK

**Figure 3:**
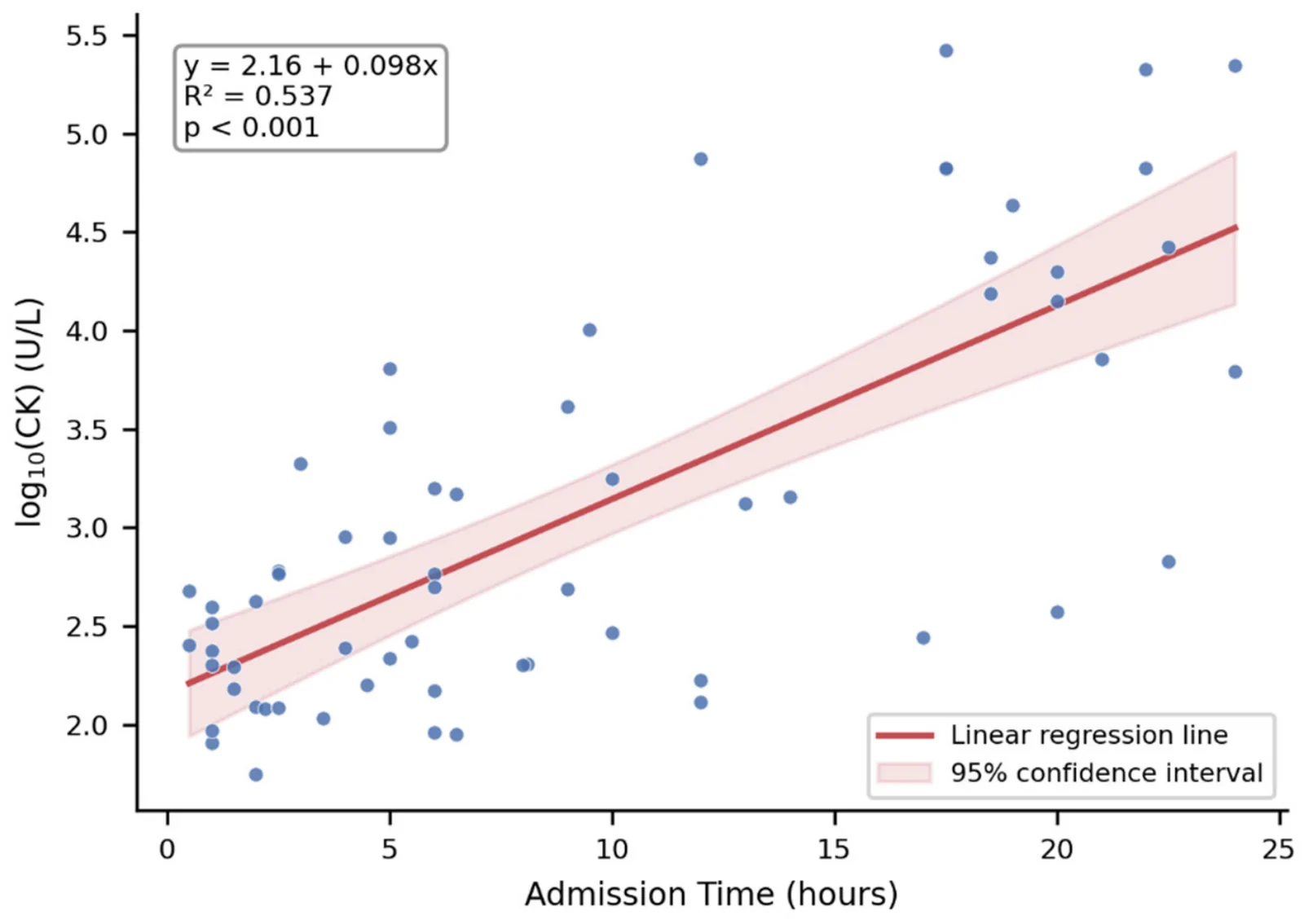
Admission Time Plotted Against Log-Transformed Serum CK Level: CK—creatine kinase; R^2^—coefficient of determination (share of variance the model accounts for); log10—base-10 logarithmic transform.

A moderately strong, statistically significant positive correlation tied admission time to log-transformed CK (r = 0.733, R^2^ = 0.537, *p* < 0.001) (Figure 3).

## 4. Discussion

Within our analysis cohort of 63 earthquake-trauma patients who completed laboratory work-up, in-hospital mortality was 4.8% (3 deaths) and was associated with diminished consciousness (GCS) and elevated trauma severity indices (AIS, ISS, NISS, RTS), whereas rubble entrapment and admission time were not significantly associated with mortality. Seven additional patients who died before the diagnostic work-up could be completed were excluded; had they been included, crude in-hospital mortality among the 70 eligible patients would have been 14.3% (10/70), so the mortality of the analysis cohort should not be read as the mortality of all patients who reached our hospital. Stratifying patients by golden-hour status revealed that late presenters carried significantly heavier burdens of trauma severity, muscle-injury and inflammatory markers, and hospital stay duration; admission time correlated strongly with serum CK (r = 0.733) and predicted rhabdomyolysis with good accuracy (AUC = 0.840). Collectively, these patterns suggest that the golden hour—long central to trauma discourse [5]—may, among patients who reach hospital care, hold greater relevance in earthquake trauma for determining organ injury depth and rhabdomyolysis risk than for predicting survival itself. While the individual associations we observed (GCS and trauma severity with mortality; admission time with muscle injury) are broadly consistent with established crush syndrome pathophysiology, this study additionally provides an exploratory, threshold-based estimate (a 9-h cutoff with defined sensitivity and specificity) that, to our knowledge, has not been previously described in this population and requires external validation.

In contrast to the earthquake literature, in which rubble entrapment and crush syndrome have repeatedly been identified as mortality predictors, entrapment was not significantly associated with mortality in our analysis cohort (OR 2.00; 95% CI 0.17–23.25). This probably reflects the very small number of deaths (*n* = 3) and, importantly, the exclusion of seven entrapped patients who died before laboratory work-up, which removed the most severe entrapment-related deaths from the analysis; the wide confidence interval is compatible with both no effect and a substantial effect. Halacli and colleagues, who followed 201 intensive-care patients after the Kahramanmaraş earthquake, singled out crush syndrome presence, older age, and vasopressor dependency as independent predictors of death, alongside a protective effect for each GCS point gained [9]. For consciousness level and anatomic injury burden we observed the same directional pattern: mortality odds dropped as GCS climbed (0.61) and rose as ISS increased (1.17), supporting the view that consciousness level and anatomic injury burden are important correlates of early survival. Non-survivors also had higher AIS grades (median 4.0 vs. 2.0), although this comparison rests on only three deaths. An Adana-based intensive care cohort reached similar conclusions, tying crush syndrome and mortality predictors to trauma and clinical severity [10]. A larger series of 610 patients likewise flagged trauma severity as a leading mortality driver [11], while a separate multicenter comparison of moderate against severe-critical crush syndrome cases pointed to trauma scores and rubble-entrapment duration as key severity determinants [4].

Our data show pronounced elevations in CK, CK-MB, LDH, and CRP among patients admitted beyond the golden hour, suggesting that delayed presentation is associated with more pronounced myofibrillar damage and systemic inflammation. This pattern echoes Köroğlu et al.’s work on 33 crush syndrome patients, where entrapment exceeding six hours substantially raised acute kidney injury risk and correlated significantly with dialysis need [12]—in line with the six-hour grouping we applied. A related multicenter cohort similarly found that pre-earthquake renal function predicted mortality, with acute kidney injury occurring more frequently among patients exhibiting severe metabolic disturbance and delayed presentation [13], a pattern consistent with prior emphasis on early diagnosis in concurrent crush syndrome and renal disease [8]. Pediatric data reinforce this temporal principle: prolonged entrapment likewise amplified muscle injury markers and clinical severity among children [14], suggesting that admission timing influences clinical trajectories regardless of patient age. These observations converge with earlier work linking CK and base deficit values to extremity injury severity and outcomes [6].

From a clinical standpoint, admission time’s strong predictive performance for rhabdomyolysis—74.1% sensitivity and 80.0% specificity, with the ninth hour as the optimal cutoff—deserves attention. Güngörer and colleagues likewise flagged CK and associated lab values as meaningful, if individually insufficient, contributors to hemodialysis decisions [15]. A review by Lim examining biomarkers for rhabdomyolysis-related acute kidney injury drew a similar conclusion: peak CK alone carries limited predictive value because of its timing sensitivity and skewed distribution, though pairing it with clinical variables like admission time markedly boosts predictive strength [16]. This dovetails with earlier work casting time-dependent progression as central to muscle injury pathophysiology [1,2]. Given our moderately strong linear relationship, admission time appears to explain roughly half the variance in CK elevation, leaving the remainder to factors such as compression intensity, entrapped muscle mass, and concurrent injuries.

The more pronounced systemic inflammation we observed among late-presenting patients corroborates the prior literature. Türktan and colleagues, examining the ratios of neutrophils to lymphocytes and platelets to lymphocytes among intensive care unit (ICU)-admitted crush syndrome patients following the same disaster, found that inflammatory burden may complement other predictors of 28-day mortality [17]—a pattern consistent with the significantly elevated CRP we detected in our late-presenting group. Separately, Aydin et al. demonstrated that low Circulation, Respiration, Abdomen, Motor, and Speech (CRAMS) triage scores, used alongside conventional trauma severity measures, were associated with acute kidney injury and crush syndrome development [18], implying that straightforward clinical scoring at initial assessment could inform risk stratification even before laboratory data become available.

Debate persists regarding the golden hour’s mortality impact within general trauma populations. A four-country Asian cohort study detected no link between the interval before hospital arrival and mortality at 30 days, though functional outcomes were adversely affected [19]. Pediatric trauma data similarly revealed a non-linear prehospital time–mortality relationship, with risk shifting past a particular threshold [20]. Our finding of no significant admission-time difference between survivors and non-survivors, and of no independent association of admission time with mortality after adjustment for entrapment, is compatible with this broader pattern. It should not, however, be read as evidence that time is irrelevant to survival. Admission time, entrapment, and injury severity were interdependent in our cohort: later arrivals were far more often entrapped (24/31 vs. 8/32) and more severely injured (median ISS 9.0 vs. 4.0 and median AIS grade 2.0 vs. 1.0; Table 2), and crude mortality did not differ significantly between patients admitted before and after 6 h (2/31 vs. 1/32; *p* = 0.613). In an exploratory model adjusted for GCS, the point estimate for admission time was above 1 (adjusted OR 1.22 per hour), but its confidence interval was very wide (0.99–103.26); this illustrates that the small number of deaths precludes firm conclusions and that larger cohorts are required. Moreover, our analysis conditions on survival to hospital arrival: patients who died at the scene or during transport could not be included, and earlier arrival may reflect less severe injury, shorter entrapment, or more effective field triage rather than the benefit of earlier care itself. Our analysis also conditions on survival to the completion of laboratory work-up: the seven excluded patients were all entrapped, comatose (GCS 3), and admitted after 6 h (median 13.5 h), so they represent the most severe, late-arriving end of the spectrum, and their exclusion removes precisely the deaths most relevant to the golden-hour question and may bias the association between admission time and mortality toward the null. Such selection and time-dependent biases may distort the association between admission time and mortality in either direction, and pre-arrival interventions by emergency medical services or referring facilities were not captured. Accordingly, our data support only a limited interpretation: among patients who reached the hospital, the initial temporal window was more strongly associated with the extent of muscle injury than with early mortality, in keeping with prior calls for early diagnosis and coordinated intervention in crush syndrome management [3,7].

Only 11.1% of our cohort needed fasciotomy, and just 6.3% required dialysis—suggesting our 24-h presentation window captured a comparatively less severely injured population. Yalın and Gölgelioğlu, comparing pediatric and adult fasciotomy outcomes, found that early surgical intervention could reduce limb loss but stressed that timing should be tailored to individual clinical and laboratory findings [21]. Koyuncu and colleagues similarly reported that prompt, aggressive fluid resuscitation lowered dialysis needs [22], while Kaya and colleagues, drawing on a post-earthquake hemodialysis center’s experience, found that climbing CK levels heightened hemodialysis complication risk among patients with crush-related kidney injury [23]. Collectively, these findings underscore that the CK-time relationship we observed carries practical weight for both diagnosis and treatment-safety planning.

The recent literature indicates that time, and the classical golden hour in particular, remains relevant but functions primarily as a modulator of outcome rather than as its principal determinant. In large-scale disasters the golden hour is frequently exceeded—in our cohort, nearly half of the patients (31/63, 49.2%) arrived after 6 h—so that the patient’s physiological status at arrival and the injury burden are likely to carry greater prognostic weight than the interval from disaster to hospital arrival. Consistent with this, a single-center series of earthquake victims undergoing orthopedic surgery identified above-knee injuries and dialysis requirement as predictors of postoperative mortality, while also reporting higher mortality with delayed hospital admission [24]. Larger descriptive series of crush syndrome patients admitted through emergency departments have documented the demographics, management, and outcomes of this population [25], and in a single-center analysis of patients with earthquake-induced crush and compartment syndrome, the preoperative albumin-to-lactate ratio independently predicted mortality and was proposed as a practical early triage tool [26]. Case reports with literature review, such as that from the Marrakesh earthquake describing a patient with crush and compartment syndrome after 11 h of entrapment, illustrate the clinical picture that follows prolonged compression [27]. Taken together, these observations align with our interpretation that admission time is best regarded as a modulator of muscle and organ injury among patients who reach the hospital, and that appropriate field triage and an effective care pathway are probably more closely linked to survival than the crude interval to admission. The role of admission time in our cohort should therefore be interpreted together with these considerations rather than as a redefinition of the golden hour.

## 5. Limitations

This study has several limitations. First, admission time combined entrapment duration and transport time, which could not be analyzed separately. Because the analysis conditions on survival to hospital arrival, pre-hospital deaths, pre-arrival interventions by emergency medical services or referring facilities, and triage processes were not captured, and time-dependent survival bias cannot be excluded; the findings therefore do not permit causal inferences regarding mortality. Second, laboratory values were obtained at a single time point within the first hour of presentation, precluding assessment of temporal trajectories or peak values.

## 6. Conclusions

In this cohort of earthquake-related trauma patients who reached hospital care and completed laboratory work-up, diminished consciousness and higher trauma severity were associated with in-hospital mortality, whereas rubble entrapment and admission time were not, and admission time was more strongly associated with markers of rhabdomyolysis than with mortality. Our findings therefore suggest that, among patients who reach the hospital, the initial temporal window may modulate the extent of muscle and organ injury; they do not, however, allow us to redefine the role of the golden hour as a determinant of survival across the entire care continuum of earthquake-related trauma. The time threshold identified in this study is hypothesis-generating and requires external validation before any consideration for field triage. Rapid extraction efforts, early laboratory evaluation, and coordinated multidisciplinary response protocols remain important in mass casualty earthquake scenarios.

## Figures and Tables

**Table 1:** Baseline Sociodemographic and Clinical Characteristics by Survival Status.

Variable	Survivors (*n* = 60) Median (IQR)/*n* (%)	Non-Survivors (*n* = 3) Median (IQR)/*n* (%)	*p*
Demographic Characteristics			
Age (years)	28.0 (22.0–40.0)	35.0 (33.0–41.5)	0.333 ^a^
Male sex, *n* (%)	29/60 (48.3%)	2/3 (66.7%)	0.613 ^b^
Time to admission (hours)	6.0 (2.5–14.8)	17.5 (9.2–19.8)	0.594 ^a^
Trapped under rubble, *n* (%)	30/60 (50.0%)	2/3 (66.7%)	1.000 ^b^
Clinical Findings and Trauma Severity Scores			
GCS	15.0 (15.0–15.0)	6.0 (5.0–10.0)	***<0.001 ^a^***
AIS	2.0 (1.0–3.0)	4.0 (3.0–4.0)	***0.049 ^a^***
ISS	4.5 (1.0–10.2)	34.0 (33.5–45.5)	***0.006 ^a^***
NISS	4.5 (1.0–13.2)	34.0 (33.5–45.5)	***0.008 ^a^***
RTS	8.0 (8.0–8.0)	4.0 (4.0–5.5)	***0.002 ^a^***
Laboratory Findings			
Lactate (mmol/L)	2.9 (1.9–4.3)	5.5 (3.0–11.8)	0.488 ^a^
CK (U/L)	495.5 (199.9–4659.2)	67,000.0 (33,697.8–139,419.5)	0.096 ^a^
Creatinine (mg/dL)	0.8 (0.7–1.0)	1.4 (0.9–2.5)	0.341 ^a^
CRP (mg/L)	6.2 (2.0–14.6)	19.4 (14.9–99.1)	0.063 ^a^
Clinical Outcomes			
Rhabdomyolysis, *n* (%)	25/59 (42.4%)	2/3 (66.7%)	0.575 ^b^
Acute kidney injury, *n* (%)	3/60 (5.0%)	1/3 (33.3%)	0.181 ^b^
Dialysis (RRT), *n* (%)	2/60 (3.3%)	2/3 (66.7%)	***0.009 ^b^***
Fasciotomy, *n* (%)	6/60 (10.0%)	1/3 (33.3%)	0.302 ^b^
ICU admission, *n* (%)	17/60 (28.3%)	3/3 (100.0%)	***0.029 ^b^***
Length of hospital stay (days)	1.7 (0.0–10.3)	4.3 (2.2–14.0)	0.416 ^a^

Panel a: Mann–Whitney U test (continuous data); panel b: Fisher’s exact test (categorical data). Values shown as median [25th–75th percentile IQR] or *n* (%); bold and italic values indicate statistically significant results (*p* < 0.05). Key: IQR—Interquartile Range; GCS—Glasgow Coma Scale; AIS—Abbreviated Injury Scale; ISS—Injury Severity Score; NISS—New Injury Severity Score; RTS—Revised Trauma Score; CK—Creatine Kinase; CRP—C-Reactive Protein; RRT—Renal Replacement Therapy (dialysis); ICU—Intensive Care Unit. Laboratory values and ISS, NISS, and RTS were available for all 63 analyzed patients; the 7 non-survivors without these data were excluded (Figure 1). Because only 3 patients died, *p* values in this table should be interpreted with caution.

**Table 2:** Trauma Severity and Laboratory Parameters by Golden-Hour Admission Status.

Variable	Golden Hour (≤6 h, *n* = 32) Median (IQR)	Post-Golden Hour (>6 h, *n* = 31) Median (IQR)	*p*
Clinical Status and Trauma Scores			
GCS	15.0 (15.0–15.0)	15.0 (15.0–15.0)	0.442
AIS	1.0 (1.0–2.2)	2.0 (2.0–3.0)	***0.004***
ISS	4.0 (1.0–9.0)	9.0 (4.0–21.5)	***0.017***
NISS	4.0 (1.0–9.0)	10.0 (4.0–26.0)	***0.004***
RTS	8.0 (8.0–8.0)	8.0 (7.8–8.0)	0.965
Blood Gas and Metabolic Parameters			
Lactate (mmol/L)	2.5 (1.9–4.0)	3.2 (1.9–4.4)	0.596
pH	7.35 (7.30–7.37)	7.38 (7.27–7.41)	0.449
Base excess (mmol/L)	2.8 (1.5–5.2)	2.9 (1.9–6.2)	0.582
Urea (mg/dL)	33.5 (26.5–47.2)	45.9 (26.9–59.0)	0.213
Creatinine (mg/dL)	0.8 (0.7–1.0)	0.8 (0.6–1.1)	0.880
Muscle Injury and Inflammatory Markers			
CK (U/L)	250.1 (142.5–582.5)	6219.4 (433.0–34,927.3)	***<0.001***
CK-MB (U/L)	4.4 (2.7–10.1)	10.1 (5.5–55.1)	***0.009***
LDH (U/L)	298.5 (233.9–547.1)	768.6 (396.0–1290.1)	***0.003***
CRP (mg/L)	3.5 (1.1–7.3)	19.0 (6.6–73.6)	***<0.001***
WBC (10^3^/µL)	14.8 (11.1–17.7)	14.8 (10.5–19.4)	0.945
INR	1.0 (1.0–1.1)	1.1 (1.1–1.2)	***0.007***
Clinical Course			
Length of hospital stay (days)	0.0 (0.0–2.2)	8.7 (1.8–17.4)	***<0.001***

Mann–Whitney U test throughout, with values shown as median [25th–75th percentile IQR]. Bold and italic values indicate statistically significant results (*p* < 0.05). Key: GCS—Glasgow Coma Scale; AIS—Abbreviated Injury Scale; ISS—Injury Severity Score; NISS—New Injury Severity Score; RTS—Revised Trauma Score; CK—Creatine Kinase; CK-MB—Creatine Kinase-MB isoenzyme; LDH—Lactate Dehydrogenase; CRP—C-Reactive Protein; WBC—White Blood Cell Count; INR—International Normalized Ratio. Laboratory values and trauma scores were available for 32 patients in the ≤6-h group and 31 patients in the >6-h group (CK-MB: 31 and 22 patients, respectively); hospital stay was available for all patients.

**Table 3:** Factors Associated with In-Hospital Mortality: Univariate and Multivariable Logistic Regression.

Variable	Univariate OR (95% CI)	*p*	Model 1: Adjusted OR (95% CI)	*p*	Model 2: Adjusted OR (95% CI)	*p*	Model 3: Adjusted OR (95% CI)	*p*
Continuous variables (OR per 1-unit increase)								
Age (years)	1.02 (0.96–1.10)	0.513	–	–	–	–	–	–
Time to admission (hours)	1.08 (0.93–1.25)	0.332	1.06 (0.89–1.34)	0.528	1.22 (0.99–103.26)	0.068	1.02 (0.85–1.22)	0.850
GCS	0.61 (0.44–0.84)	***0.003***	–	–	0.55 (<0.01–0.78)	***<0.001***	–	–
ISS	1.17 (1.02–1.35)	***0.022***	–	–	–	–	1.12 (1.04–1.29)	***0.001***
Categorical variables								
Trapped under rubble	2.00 (0.17–23.25)	0.580	1.02 (0.02–18.70)	0.988	–	–	–	–
Rhabdomyolysis	2.72 (0.23–31.69)	0.424	–	–	–	–	–	–
Acute kidney injury	9.50 (0.66–136.70)	0.098	–	–	–	–	–	–

Univariate estimates: maximum-likelihood logistic regression with Wald 95% confidence intervals. Multivariable estimates: Firth penalized-likelihood logistic regression with profile penalized-likelihood 95% confidence intervals and *p* values. Model 1 included admission time (per hour) and rubble entrapment; Model 2 included admission time (per hour) and GCS (per point). Model 3 included admission time (per hour) and ISS (per point). With only three deaths among the 63 analyzed patients, all multivariable estimates are imprecise and exploratory (Models 1 and 2 show near-complete separation). Bold and italic values indicate statistically significant results (*p* < 0.05); a dash indicates that the variable was not included in the model. Key: OR—Odds Ratio; CI—Confidence Interval; GCS—Glasgow Coma Scale; ISS—Injury Severity Score.

## Data Availability

The original contributions presented in this study are included in the article. Further inquiries can be directed to the corresponding author.

## Abbreviations

AIS, Abbreviated Injury Scale; AUC, Area Under the Curve; CI, Confidence Interval; CK, Creatine Kinase; CK-MB, Creatine Kinase-MB isoenzyme; CRP, C-Reactive Protein; GCS, Glasgow Coma Scale; ICU, Intensive Care Unit; INR, International Normalized Ratio; ISS, Injury Severity Score; LDH, Lactate Dehydrogenase; NISS, New Injury Severity Score; OR, Odds Ratio; RRT, Renal Replacement Therapy; ROC, Receiver Operating Characteristic; RTS, Revised Trauma Score; WBC, White Blood Cell count.

## References

[1] Usuda D., Shimozawa S., Takami H., Kako Y., Sakamoto T., Shimazaki J., Inoue J., Nakayama S., Koido Y., Oba J. (2023). Crush syndrome: A review for prehospital providers and emergency clinicians. J. Transl. Med..

[2] Luo Y., Liu C., Li D., Yang B., Shi J., Guo X., Fan H., Lv Q. (2023). Progress in the diagnostic and predictive evaluation of crush syndrome. Diagnostics.

[3] Bağiş K.O., Durmuş S., Ekşi A., Gümüşsoy S., Kekreli Göylüsün H., Çakaloğlu T., Gümüş M., Erik E. (2026). Barriers delaying treatment in cases at risk of crush syndrome in major disasters: Kahramanmaraş earthquake experience. J. Nephrol..

[4] Turgutalp K., Ozturk S., Koc N.S., Dolarslan M.E., Kocyigit I., Turgut D., Sahutoglu T., Torun D., Usalan O., Altunoren O. (2026). Impact of crush syndrome severity on clinical outcomes after the earthquakes in southeastern Türkiye. BMC Nephrol..

[5] Alarhayem A.Q., Myers J.G., Dent D., Liao L., Muir M., Mueller D., Nicholson S., Cestero R., Johnson M., Stewart R. (2016). Time is the enemy: Mortality in trauma patients with hemorrhage from torso injury occurs long before the “golden hour”. Am. J. Surg..

[6] Gur A., Simsek Y. (2024). The impact of creatine kinase and base excess on the clinical outcome of crush injuries sustained during the Kahramanmaras/Turkey earthquakes on February 6, 2023. Medicine.

[7] Turgutalp K., Kıykım A.A., Oto Ö.A., Demir S., Çobanoğlu D., Oğuz İ., Ünver R.E., Şen İ.A., Özer C., Sever M.Ş. (2024). Analysis of crush syndrome patients with and without acute kidney injury during the 2023 Kahramanmaraş earthquake: Experience of a tertiary referral center from Türkiye. Turk. J. Nephrol..

[8] Ozturk S., Tuglular S., Olmaz R., Kocyigit I., Kibar M.U., Turgutalp K., Torun D., Sahutoglu T., Usalan O., Gungor O. (2024). Patients with crush syndrome and kidney disease: Lessons learned from the earthquake in Kahramanmaraş, Türkiye. Kidney Int..

[9] Halacli B., Guven G., Kaya E.K., Yildirim M., Bulut Yuksel N.D., Kocak G., Bulut K., Gulen D., Sullu B.E., Kilicaslan B. (2025). Evaluation of the clinical characteristics and outcomes of patients admitted to intensive care units after the Kahramanmaras (Türkiye) earthquake: A multicenter observational study. Front Med..

[10] Kara Ü., Arslan B., Kaya Özdoğan H., Gülek Gürbüz K., Ünlü N., Boğa Z. (2026). Crush syndrome and mortality predictors in intensive care unit patients after earthquakes. Signa Vitae.

[11] Onan E., Torun D., Kozanoğlu R., Miçözkadıoğlu H., Beyaz S., Özgözen L., Turgut N., Demiroğlu Y.Z., Karagün Ö., Ergenoğlu P. (2024). Mortality factors in crush syndrome. Ulus. Travma Acil Cerrahi Derg..

[12] Köroğlu M., Karakaplan M., Barakat M., Ergen E., Aslantürk O., Özdeş H.U., Bıçakcıoğlu M., Yaşar Ş. (2024). Predictive factors for acute kidney injury and amputation in crush injuries from the Kahramanmaraş earthquakes. Ulus. Travma Acil Cerrahi Derg..

[13] Daniş R., Öztürk S., Koçyiğit İ., Kılıç J., Civan M., Şahutoğlu T., Torun D., Onan E., Kıykım A.A., Cevher S.K. (2025). Pre-earthquake kidney function is a predictor of outcomes in earthquake-related crush syndrome. BMC Nephrol..

[14] Zırhlı Selçuk Ş., Elmas A.T., Tabel Y. (2024). Crush syndrome of children in Kahramanmaraş earthquake: A single center experience in Malatya. Turk. Arch. Pediatr..

[15] Güngörer B., Demirtaş B., Kundi H., Özgürlük İ., Ocak M. (2025). iCEBc: A new predictor for hemodialysis need in patients with crush injury—Experience from the February 6, 2023, Turkey earthquake. Hong. Kong J. Emerg. Med..

[16] Lim A.K.H. (2025). Predicting acute kidney injury in acute rhabdomyolysis. J. Clin. Med..

[17] Türktan M., Doğan Ö., Gök M.G., Aydın K., Güleç E., Hatipoğlu Z., Arslan Y.K., Özcengiz D. (2024). Mortality risk factors for crush syndrome after an earthquake in Türkiye: Do systemic inflammatory parameters play any role?. Ulus. Travma Acil Cerrahi Derg..

[18] Aydin K., Ozel Yesilyurt A., Cetinkaya F., Gok M.G., Dogan O., Ozcengiz D. (2024). Earthquake victims in focus: A cross-sectional examination of trauma and management in intensive care unit. BMC Emerg. Med..

[19] Chen C.H., Shin S.D., Sun J.T., Jamaluddin S.F., Tanaka H., Song K.J., Kajino K., Kimura A., Huang E.P.-C., Hsieh M.-J. (2020). Association between prehospital time and outcome of trauma patients in 4 Asian countries: A cross-national, multicenter cohort study. PLoS Med..

[20] Rickenbach O.N., Aldridge J., Tumin D., Greene E., Ledoux M., Longshore S. (2024). Prehospital time and mortality in pediatric trauma. Pediatr. Surg. Int..

[21] Yalın M., Gölgelioğlu F. (2023). A comparative analysis of fasciotomy results in children and adults affected by crush-induced acute kidney injury following the Kahramanmaraş earthquakes. Medicina.

[22] Koyuncu S., Sipahioglu H., Bol O., İlik H.K.Z., Dilci A., Elmaağaç M., Yalçınkaya M., Gencer V., Ozan F., Günal A.I. (2023). The evaluation of different treatment approaches in patients with earthquake-related crush syndrome. Cureus.

[23] Kaya B., Balal M., Seyrek N., Mete B., Karayaylali I. (2024). Hemodialysis experience after Kahramanmaraş earthquake. J. Clin. Med..

[24] Erdem E.H., Aydinli B., Dogru S., Ozmen H., Atilla M., Yildirim I. (2025). Postoperative outcomes in earthquake victims with orthopedic trauma: A focus on mortality and dialysis needs. BMC Anesthesiol..

[25] Görmeli Kurt N., Elmas Akgün F., Mutlu R.İ., Unutmaz M., Çamcı M. (2024). A retrospective study of 377 patients admitted as an emergency with crush syndrome after the Türkiye–Syria earthquakes. Med. Sci. Monit..

[26] Taygurt T.N., Erdem G., Taygurt S., Koç M.N. (2026). Anesthetic management and early outcomes in patients with earthquake-induced crush and compartment syndrome: A single-center retrospective analysis. Ulus. Travma Acil Cerrahi Derg..

[27] Zidouh A., Tebib T., Salmi O., Mourouth H., Ziadi A. (2025). Crush syndrome after the Marrakesh earthquake: A case report and review of the literature. Oxf. Med. Case Rep..

